# The harmonies played by miR-302/367 cluster in pluripotency, reprogramming, and rejuvenation

**DOI:** 10.1093/stcltm/szaf072

**Published:** 2026-02-15

**Authors:** Melika Zamanian, Sharif Moradi, Hossein Baharvand

**Affiliations:** Department of Applied Cell Sciences, Faculty of Basic Sciences and Advanced Medical Technologies, Royan Institute, ACECR, Tehran 1665659911, Iran; Department of Stem Cells and Developmental Biology, Cell Science Research Center, Royan Institute for Stem Cell Biology and Technology, ACECR, Tehran 1665659911, Iran; Department of Stem Cells and Developmental Biology, Cell Science Research Center, Royan Institute for Stem Cell Biology and Technology, ACECR, Tehran 1665659911, Iran; Department of Developmental Biology, School of Basic Sciences and Advanced Technologies in Biology, University of Science and Culture, Tehran 1461968151, Iran; Department of Stem Cells and Developmental Biology, Cell Science Research Center, Royan Institute for Stem Cell Biology and Technology, ACECR, Tehran 1665659911, Iran; Department of Developmental Biology, School of Basic Sciences and Advanced Technologies in Biology, University of Science and Culture, Tehran 1461968151, Iran

**Keywords:** microRNA, senescence, cell fate conversion, iPSC, ESC, regenerative medicine

## Abstract

The conserved, pluripotency-associated miR-302/367 cluster coordinates cell fate and aging via epigenetic, cell cycle, and signaling regulation. Highly expressed in pluripotent stem cells and silenced during differentiation, it promotes efficient somatic cell reprogramming by suppressing senescence mediators (eg, p16INK4a, p21) and replacing oncogenes such as c-Myc to minimize tumorigenic risks. Beyond pluripotency, the miR-302/367 cluster reduces oxidative stress, mitochondrial dysfunction, and fibrosis, indicating therapeutic potential in age-associated conditions such as neurodegenerative, ocular, and fibrotic diseases. This review summarizes the dual ability of miR-302/367 cluster in promoting cell state transitions and transiently resetting cellular aging to enable healthspan extension. We critically discuss the pivotal role of miR-302/367 cluster in pluripotency and reprogramming while countering aging hallmarks. Finally, we explore how combining single-miRNA therapeutics with clinically viable delivery systems (lipid nanoparticles and extracellular vesicles) can link cellular reprogramming with targeted rejuvenation therapies.

## Graphical Abstract

**Graphical Abstract:**
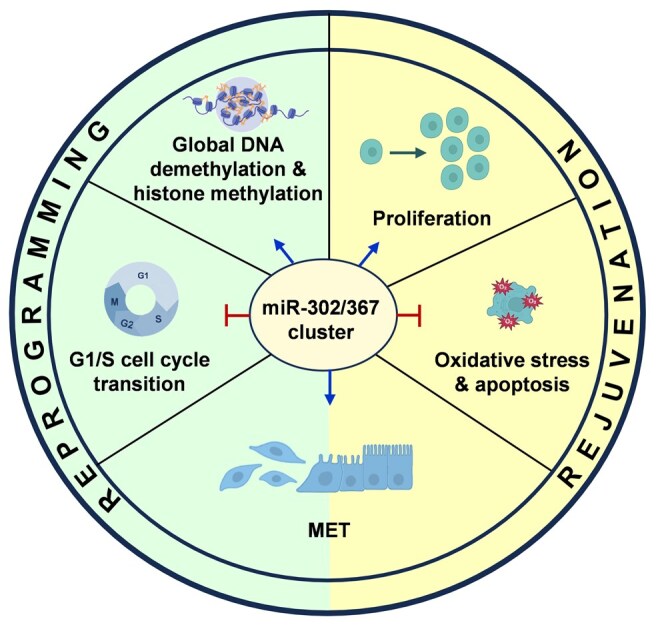

Significance statement A major challenge in regenerative medicine is finding ways to safely restore the body’s natural capacity for repair, particularly in aged tissues. This review highlights a powerful molecular tool, the miR-302/367 cluster, which acts as a master conductor of cell identity. It offers a dual therapeutic strategy: it can reprogram adult cells into versatile stem cells for therapy, while also directly reversing aging processes in damaged tissues. By bridging the gap between stem cell biology and aging research, our review outlines how this single family of microRNAs could unlock new approaches for treating degenerative diseases and extending healthy lifespan.

## Introduction

MicroRNAs (miRNAs) are small, noncoding RNAs that control gene expression at the post-transcriptional level, functioning as major regulators of cellular identity, plasticity, and homeostasis. By forming the RNA-induced silencing complex (RISC) with Argonaute proteins, miRNAs suppress translation or degrade target mRNAs, enabling them to coordinately modulate tens to hundreds of transcripts. This establishes miRNAs as pivotal players in fine-tuning gene regulatory networks that govern development, disease, and—critically—aging.^1–5^ Among the ∼1600 miRNAs in the human genome, the miR-302/367 cluster stands out for its dual role in pluripotency maintenance and cellular rejuvenation, bridging stem cell biology and aging research.^6–8^

Embryonic stem cells (ESCs) harbor a unique miRNA signature, including the miR-302/367 cluster, miR-290∼295 cluster (mouse), and the chromosome 19 miRNA cluster known as C19MC (human), which are tightly regulated by core pluripotency-associated transcription factors (Oct4, Sox2, Nanog) and silenced upon differentiation.^9–12^ During somatic cell reprogramming to pluripotency, ESC-enriched miRNAs (eg, miR-302/367 cluster) are upregulated while somatic cell-enriched miRNAs are downregulated. In contrast, upon exit from pluripotency, differentiation-associated miRNAs (eg, let-7 and miR-145 families) are upregulated, driving cells toward specialized fates.^13^ This dynamics is recapitulated over the induced pluripotent stem cell (iPSC) generation, where an increase in reprogramming efficiency occurs upon suppression of somatic cell–affiliated miRNAs such as miR-21 and miR-29a and overexpression of ESC-specific miRNAs particularly miR-302/367 cluster. The latter substantially promotes the generation of iPSCs, and its overexpression alone may even replace Yamanaka factors, underscoring its ability in resetting cellular aging clocks.^14–19^ Notably, the cluster’s ability to bypass senescence—by suppressing *p21* and modulating TGF-*β* signaling—suggests its broader role in antagonizing age-related decline.^20–22^

The aging process is orchestrated by the progressive deterioration of epigenetic and transcriptional programs. Within this complex landscape, miRNAs have emerged as highly important regulators; while certain miRNAs such as miR-34a-5p and miR-128a-3p promote cellular senescence, others such as the miR-302/367 cluster show remarkable rejuvenation potential, offering a viable therapeutic strategy for age-related decline. Mechanistically, miR-34a-5p promotes p53-mediated senescence by inhibiting *SIRT1*, whereas miR-302a-3p inhibits RasG12V-induced senescence by targeting *p21.*^21^^,^^23–25^ Notably, an inducible vector encoding miR-302a, -302 b, -302c, and -302d was shown to significantly contribute to the epigenetic rejuvenation of somatic cells by targeting the epigenetic regulators AOF1, LSD1, MECP1-p66, and MECP2.^18^ The miR-302/367 cluster further mitigates aging hallmarks—fibrosis, neurodegeneration, and metabolic dysfunction—via cell cycle regulation, suppression of epithelial-to-mesenchymal transition (EMT), and TGF-*β* pathway modulation.^26–29^ These findings highlight miR-302-367 cluster as a molecular hub linking pluripotency and longevity.

Despite its well-documented role in pluripotency and reprogramming, the potential of miR-302/367 cluster to reverse age-associated pathologies remains underexplored. In this review, we summarize current knowledge on the miR-302/367 cluster, focusing on its critically important roles in pluripotency, reprogramming, and rejuvenation. By dissecting its mechanisms across these contexts, we highlight its great promise as a therapeutic tool for regenerative medicine purposes and age-intervention strategies.

## The miR-302/367 cluster: biogenesis, regulation, and functional insights

The miR-302/367 cluster is embedded in the eighth intron of the *LARP7* gene on chromosome 4 and contains five distinct members, including miR-302a, -302 b, -302c, -302d, and miR-367, each with their characteristic -5p and -3p species. The seed sequence of miR-367 is slightly different from that of the other members. However, it cooperatively silences mRNA targets with other members of the cluster to help maintain pluripotency and suppress aging-related pathways.^10^^,^^26^ The miR-302/367 cluster is highly conserved across vertebrates, with variability in copy number and genomic location reflecting evolutionary adaptations. The 3ʹ arm of miR-302 miRNAs shows striking conservation and dominant expression, underscoring its crucial role in pluripotency and stemness.^30^ As with most other miRNAs, the miR-302/367 cluster is transcribed by RNA polymerase II as a polycistronic pri-miRNA. The resulting pri-miRNA is processed by the DROSHA-DGCR8 (microprocessor) complex into pre-miRNAs, which are then exported to the cytoplasm via Exportin-5. Next, the RNase III enzyme DICER further processes the pre-miRNA molecules (loop removal and 3ʹ overhang generation) and produces mature miRNA duplexes, with the guide strand incorporated into RISC to silence target genes through translational repression or cleavage of the target mRNA.^4^^,^^16^^,^^20^

**Figure 1.**
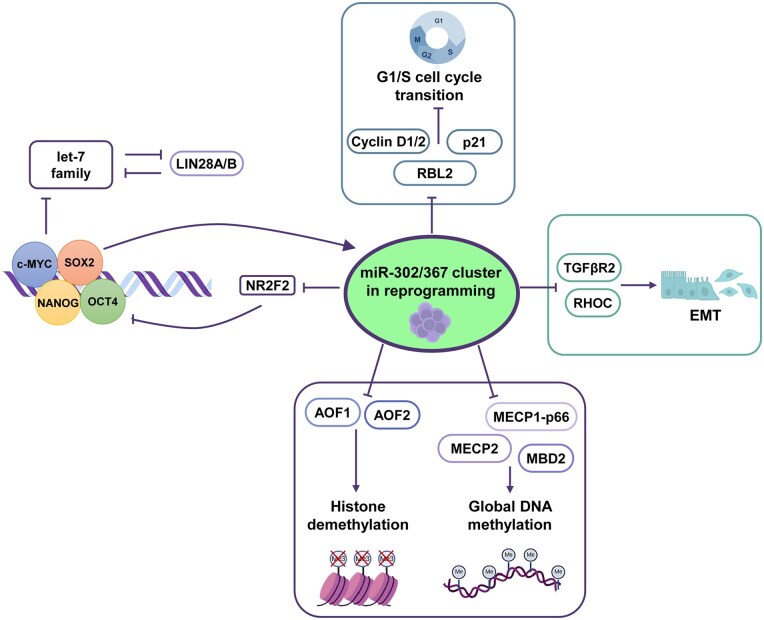
Mechanisms underlying the function of the miR-302/376 cluster during the generation and maintenance of PSCs. The mechanisms through which the miR-302/376 cluster regulates the induction and maintenance of pluripotency involve the modulation of gene expression across three main pathways: cell cycle regulation, chromatin remodeling, and TGF-β signaling.

The expression of miR-302/367 cluster is tightly regulated by major pluripotency factors (Oct4, Sox2, Nanog) and epigenetic-related mechanisms.^9^ In differentiated cells, CpG methylation and repressive histone marks (eg, H3K9me and H3K27me3) promote silencing of the miRNA cluster, while permissive histone marks (eg, H3K4me3) activate it in undifferentiated PSCs.^31^^,^^32^ As shown in Figure 1, the miR-302/367 cluster serves diverse functions in stem cell pluripotency and somatic cell reprogramming, tightly regulates cell cycle progression, modulates epigenetic processes, and promotes mesenchymal-to-epithelial transition (MET). In addition to supporting pluripotency, the miR-302/367 cluster drives cellular rejuvenation by suppressing senescence (via p21 inhibition and TGF-β pathway modulation) and promoting pluripotent reprogramming (via inhibition of certain epigenetic regulators).^18^^,^^21^^,^^28^ These findings highlight the indispensable role of miR-302/367 cluster in supporting stemness and resisting aging. This dual capacity to suppress differentiation-associated processes and activate pluripotency pathways explains why the miR-302/367 cluster, or individual members thereof, has become a cornerstone of reprogramming strategies, enabling efficient iPSC generation and aging reversal both in combination with other factors and as a standalone agent.

## The miR-302/367 cluster in pluripotency and cell fate reprogramming

The miRNA biogenesis pathway is indispensable for pluripotency induction and maintenance, as evidenced by severe defects in *Dicer* or *Dgcr8* knockout models, which impair PSC proliferation, differentiation, and teratoma or chimera formation.^33^^,^^34^ Highly expressed in ESCs and iPSCs, the miR-302/367 cluster—alongside the miR-290 (mouse) and miR-371-373 (human) clusters—shares the conserved AAGUGC seed sequence characteristic of ESC-specific cell cycle–regulating (ESCC) miRNAs. These miRNAs sustain self-renewal and are silenced upon differentiation.^10^^,^^35^ miR-302 family members are upregulated in the ground state of pluripotency (ie, in 2i- or R2i-cultured ESCs),^36^ which is characterized by minimal differentiation leakage.^37^ This finding suggests that miR-302 miRNAs contribute to maintaining ground-state ESCs by suppressing differentiation processes. In line with this, ESCC miRNAs, including miR-302 family members, were reported to oppose the pro-differentiation functions of the let-7 family of miRNAs,^38^ thereby enabling the unique cell cycle properties of ESCs. Moreover, the overexpression of miR-302b-3p, a representative member of this cluster, partially restores the viability to ground-state ESCs deprived of R2i chemicals, further supporting its pivotal role in ESC maintenance even in the absence of exogenous differentiation-suppressing compounds.^39^ We have also demonstrated that overexpression of miR-302b-3p potently stimulates ESC self-renewal and can functionally replace exogenous leukemia inhibitory factor (LIF).^39^ Since LIF is known to inhibit neural differentiation in ESCs,^40^ miR-302b-3p and/or other family members may similarly serve to repress neural differentiation in LIF-withdrawn ESCs. Supporting this hypothesis, miR-302a has been reported to play a major role in suppressing the induction of the neural ectoderm by targeting NR2F2 (also known as NRF2), an inhibitor of OCT4 that is activated during the early neural differentiation of ESCs.^41^^,^^42^ During the inner cell mass (ICM)-to-ESC transition, miR-302b-3p substantially increased the efficiency of ESC line establishment.^43^ This effect can be explained, at least in part, by the targeting of specific differentiation pathways by miR-302b-3p, thereby allowing self-renewal to be established in ICM outgrowths transitioning into ESCs.

Notably, the miR-302/367 cluster alone can replace exogenous transcription factors during pluripotent reprogramming. Anokye-Danso et al.^19^ reported that viral overexpression of the miR-302/367 cluster enabled the generation of mouse and human iPSCs in the absence of conventional reprogramming factor. Although this report has not been independently replicated, the fact that numerous studies have demonstrated that the miR-302/367 cluster or individual members from it remarkably enhance the efficiency of iPSC reprogramming from somatic cells^16–18^ highlights its role as a key reprogramming agent in induced pluripotency. In support of these data, it has been shown that CRISPR-mediated activation of the cluster’s promoter has been shown to enhance iPSC generation.^44^ Additionally, a recent study demonstrated that both the miR-290 and miR-302/367 clusters were essential for reprogramming mouse fibroblasts to iPSCs, as the simultaneous loss of the two clusters, but not the loss of either cluster alone, completely prevented iPSC formation. This suggests a functional redundancy between these two ESCC miRNA clusters, where the activity of one cluster compensates, at least partially, for the lack of the other. This functional redundancy may explain why a single allele of the cluster sufficed to maintain pluripotency in human iPSCs.^45^^,^^46^ The miR-302/367 cluster also mitigates tumorigenic risks and can substitute for the oncogenic transcription factor c-Myc in partial reprogramming protocols.^16^ Specifically, the miRNAs miR-302a, -302 b, -302c, and -302d balance pluripotency and tumor suppression by silencing oncogenic processes (eg, targeting BMI-1 and AKT1) and inhibiting CDK2 and CDK4/6-related cell cycle pathways.^47^^,^^48^ Thus, the miR-302/367 cluster fine-tunes cell cycle regulation to balance pluripotency and tumor suppression, offering a safer route for clinical iPSC applications in regenerative medicine.

Mechanistically, the miR-302/367 cluster supports iPSC reprogramming and ESC self-renewal through three critical pathways (Figure 1): (1) Cell cycle acceleration via suppression of G1/S inhibitors (eg, p21, RBL2), as well as the repression of CYCLIN D1 and Cyclin D2^20^^,^^35^^,^^48–51^; (2) Epigenetic remodeling by targeting methyl-CpG binding proteins 1/2 (MECP1/2, MBD2) and histone demethylases (AOF1/2)^18^^,^^52–54^; and (3) MET induction through TGF-β Receptor2 (TGF-βR2) inhibition and E-cadherin upregulation.^16^^,^^55^ The diversity of cellular processes and signaling pathways co-regulated by miR-302/367 cluster members demonstrates that a strong driver of ESC/iPSC generation and maintenance must be a multifunctional regulatory player capable of simultaneously modulating multiple critical pathways.

Although the miR-302/367 cluster is important for maintaining pluripotency, its regulatory potential has also been established in certain progenitor cells,^56–58^ suggesting a broader role in tissue regeneration. For instance, intravenous administration of miR-302b/c-3p in mice promotes regeneration of tissue damaged by *Streptococcus pneumoniae* by stimulating the proliferation of local epithelial progenitor cells via downregulation of *p21.*^57^ This finding is further supported by evidence demonstrating its pivotal role in cellular transdifferentiation, also known as lineage reprogramming. As summarized in Table 1, this cluster reprograms astrocytes to neuroblasts or oligodendrocyte progenitors (when combined with valproic acid, VPA), improving functional recovery in demyelination models.^60–63^ Similarly, miR-302a-3p alone efficiently converts fibroblasts into induced neural stem cells (iNSCs), while its combination with specific transcription factors reprograms hepatocytes to functional insulin-producing islet-like cells.^63^ These effects are primarily mediated through modulation of BMP and TGF-β/Activin/Nodal signaling pathways, which critically maintain the equilibrium between pluripotency and differentiation.^64^^,^^65^ Collectively, these findings establish the miR-302/367 cluster as a potent regulator of cell state reprogramming with substantial therapeutic potential in regenerative medicine.

**Table 1.** The functions of miR-302/367 cluster during cell fate reprogramming.

	miRNA ID	miRNA delivery method	Starting cell type	Reprogramming factors/agents	Function of miRNA	Target of miRNA	Reference
Pluripotent reprogramming	miR-200c-3p, miR-302a-, b-, c-, d-3p, miR-369 -3p, & miR-369-5p	Transient transfections	mASC, hASC & HDF	No reprograming factors used	Generation of iPSCs independent of OSKM	*Aof1^a^*	Miyoshi et al.^17^
	miR-302a-, b-, c-, d-3p	Transient transfection	hHFC	No reprograming factors used	Induction of global genomic demethylation	*AOF1*, *AOF2*, *MECP1-p66,* & *MECP2*	Lin et al.^18^
	miR-302/367 cluster	Lentiviral transduction	MEF & HDF	VPA	Yielding higher reprogramming efficiency than OSKM	ND	Anokye-Danso et al.^19^
	miR-302b-3p	Transient transfection	HFF & HLF	OSKM	Acceleration of MET	*RHOC* & *TGFβR2*	Subramanyam et al.^52^
	miR-302a-, b-, c-, d-3p	Transient transfection	hASC	OSKM	Increased reprogramming efficiency	*NR2F2*	Hu et al.^42^
	miR-302a-, b-, c-, d-3p	Lentiviral transduction	hUCB	OSKM	NANOG upregulation & increased conversion of partially to fully reprogrammed iPSCs	*MBD2*	Lee et al.^53^
	miR-302/367 cluster	Transient transfection	HDFn	OSKM+ NANOG & LIN28A	Increased reprogramming efficiency	ND	Kogut et al.^15^
	miR-302/367 cluster	Retroviral transduction	MEF	OSK	Acceleration of MET by increasing E-cadherin expression	*TGFβR2*	Liao et al.^16^
	Knockout of miR-290/295 & miR-302/367 clusters	NA	MEF	OSK	Double knockouts suppressed iPSC generation	ND	Ye et al.^46^
	miR-302/367 cluster	Transient transfection	hUC	Episomal OSK, episomal SV40LT + Small molecules (A-83-01, CHIR99021, Tzv, sodium butyrate, cyclic pifithrin-a, & PD0325901)	Increased reprogramming efficiency	ND	Li et al.^59^
	Activation of miR-302/367 cluster with CRISPRa	NA	hLCL	OSK, L-MYC, LIN28A, p53 shRNA, & NaB	Enhanced reprogramming efficiency and decreased heterogeneity	ND	Sokka et al.^44^
Lineage reprogramming (trans-differentiation)	miR-302/367 cluster	Lentiviral transduction	Astrocytes	VPA	Increased myelin regeneration	ND	Ghasemi‐Kasman et al.^60^
	miR-302/367 cluster	Lentiviral transduction	Astrocytes	VPA	Induction of neuroblasts and neurons from astrocytes	ND	Ghasemi‐Kasman et al.^61^
	miR-302a-3p	Lentiviral and adenoviral transduction	HFF/HDF and mouse L929 fibroblast cell line	VPA and vitamin C	Rapid and efficient generation of iNSCs	ND	Li et al.^62^
	miR-302a-3p	Transient transfection	Human adult hepatocytes (HL-7702 cell line)	*Pdx1*, *Ngn3,* and *MafA*	Facilitated production of pancreatic islets-like cells	ND	Lu et al.^63^

Abbreviations: hHFC: Human hair follicle cell; mASC & hASC: Mouse and human Adipose stromal cell; VPA: Valproic acid; HDF: Human dermal fibroblast; MEF: Mouse embryonic fibroblast; ND: Non-determined; HFF: Human foreskin fibroblast; HLF: Human lung fibroblast; TGFβR2: TGF-β receptor2; hUCB: Human umbilical cord blood; HDFn: Human primary neonatal fibroblast; NA: not applicable; MET: mesenchymal-to-epithelial transition; hUC: Human urine-derived cell; NaB; Sodium butyrate; hLCL: Human lymphoblastoid cell line; NSC: Neural stem cell.

a miRNA target predicted or only partially validated.

## The miR-302/367 cluster: a stimulator of cellular rejuvenation

### Contribution of miRNAs to the regulation of aging

Aging represents a complex biological phenomenon characterized by the gradual deterioration of physiological functions and loss of tissue homeostasis, leading to increased susceptibility to chronic conditions, including neurodegenerative diseases, cardiovascular disorders, cancer, diabetes, and age-related vision impairment. miRNAs have emerged as important regulators of these processes, with specific miRNA families (eg, miR-17-92 cluster, let-7 miRNAs, and miR-34a-5p) being differentially expressed across aging tissues and shown to directly regulate longevity pathways in model organisms.^66–68^ Importantly, age-associated alterations in miRNA expression profiles have been shown to affect fundamental pathways governing cellular senescence (miR-34a-5p), oxidative stress responses (miR-200 family), and inflammatory cascades (miR-146a-5p).^69^^,^^70^ Notably, circulating miRNA signatures (eg, elevated miR-21 and decreased miR-126) have now been identified as biomarkers of biological aging, with higher predictive value than chronological age alone.^71^^,^^72^

miRNAs also regulate the self-renewal properties in stem cells as they undergo functional decline due to increasing exposure to both endogenous and environmental stressors. The imbalance between cellular damage and self-renewal mechanisms ultimately contributes to systemic dysfunction and disease progression. This decline is characterized by typical shifts in miRNA profiles—notably the downregulation of pluripotency-associated miRNAs (eg, miR-302/367 and miR-290/295 clusters) and upregulation of senescence-promoting miRNAs (eg, miR-34a-5p and miR-146a-5p)—that drive stem cell exhaustion.^69^^,^^73^

Investigations into key aging hallmarks, including genomic instability (regulated by miR-155-5p), telomere shortening (modulated by the miR-290/302 seed family), epigenetic dysregulation (miR-29 family), mitochondrial dysfunction (miR-181 family), and cellular senescence (miR-302/367 cluster), provide valuable insights for developing targeted anti-aging strategies using a miRNA-based approach.^74–77^ Within this context, the miR-302/367 cluster has emerged as a particularly promising therapeutic target due to its multifaceted roles in suppressing age-related cellular decline, serving as a molecular nexus between pluripotency maintenance and senescence resistance.^21^^,^^26^^,^^27^

### miR-302/367 cluster’s anti-senescence functions in neurodegenerative and eye disorders

**Figure 2.**
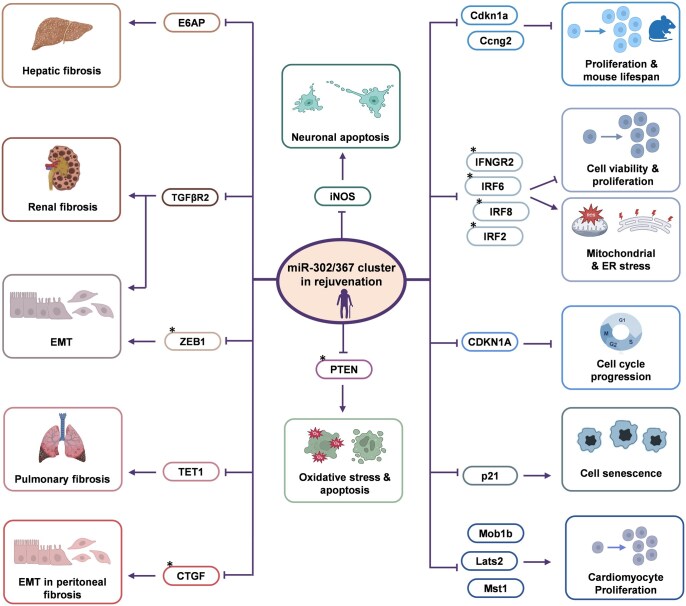
Contribution of the miR-302/376 cluster to rejuvenation. The miR-302/367 cluster plays a key role in opposing the process of aging across various models, including Alzheimer’s disease, ocular conditions, fibrosis, and cellular senescence. An asterisk (*) denotes a miRNA target that has not been fully verified.

Several studies have revealed the considerable capacity of the miR-302/367 cluster to modulate aging and rejuvenation processes (Figure 2, Table 2), with particularly promising implications for neurodegenerative disorders such as Alzheimer’s disease (AD) and Parkinson’s disease (PD). In AD models, the miR-302/367 cluster shows neuroprotective effects through its inhibition of *PTEN* within the Akt signaling pathway, resulting in subsequent upregulation of Nrf2 and HO-1 that ameliorate amyloid-β-induced neurotoxicity.^78^ Clinical findings show that the expression of the *LARP7* gene, the host gene of the miR-302/367 cluster, is significantly reduced in peripheral blood cells of AD patients, suggesting its potential utility as a novel diagnostic biomarker.^78^ Furthermore, therapeutic administration of the miR-302/367 cluster in combination with VPA has shown significant efficacy in improving cognitive function and repairing neural damage in streptozotocin-induced AD animal models.^79^ Since the combination of the miR-302/367 cluster and VPA has been reported to efficiently induce iPSC generation from mouse and human somatic cells,^19^ this raises the question of why this same combination did not promote in vivo iPSC generation and/or teratoma formation from any potentially emerging pluripotent cells. The most likely explanation is that the complete induction of pluripotency in vivo may be inhibited due to the non-permissive microenvironment of adult tissues. Furthermore, even iPSC derivation in vitro requires exogenous bFGF (human) or LIF (mouse) (numerous references). Moreover, although in vivo OSKM overexpression led to numerous tumors in mice,^92^ the observation that the miR-302/367 cluster exerts anti-tumor effects beyond a specific threshold^18^ may explain the absence of tumors following in vivo administration of the miR-302-VPA combination. In PD models, ectopic expression of miR-302b-5p has been demonstrated to reduce neuronal apoptosis and enhance cell viability through its interaction with inducible nitric oxide synthase (iNOS), thereby potentiating the neuroprotective effects of insulin-like growth factor-1 (IGF-1).^80^ Interestingly, the -5p arm of miR-302b is not the dominant strand in PSCs, and in most other cellular contexts, where the -3p arm of this miRNA (ie, miR-302b-3p) is incorporated into the RISC as the guide strand to exert regulatory effects. The observation that miR-302b-5p, as the passenger strand with a sequence distinct from the -3p strand, plays an important role in reducing neural aging suggests that arm switching in miR-302b (and possibly other miR-302/367 family members) may be a more common phenomenon, enabling more diverse functions for these miRNAs. These collective findings establish the miR-302/367 cluster as a highly promising therapeutic candidate for neurodegenerative conditions.

**Table 2.** The function of miR-302/367 cluster during rejuvenation.

	miRNA ID	miRNA delivery method	In vitro or in vivo model	Function of miRNA	Target of miRNA	Reference
Aging-associated neurodegeneration	miR-302/367 cluster	Transient transfection	Human neuroblastoma SK-N-MC cells, induced by Aβ	Attenuated Aβ-induced oxidative stress and apoptosis	*PTEN* ^a^	Li et al.^78^
	miR-302/367 cluster	Lentiviral transduction	Intracerebroventricular STZ injection in mice	Increased neural repair	ND	Ghasemi-Kasman et al.^79^
	miR-302b-5p	Transient transfection	*In vitro:* SH-SY5Y cells, induced by MPTP *In vivo:* MPTP-induced PD mouse model	Enhanced the neuroprotective effect of IGF-1	*iNOS*	Cui et al.^80^
Age-related ocular disease	miR-302a-5p	Transient transfection	*In vitro:* hCEC, induced senescence by IFN-γ *In vivo:* Cryo-injured corneal endothelium	*In vitro:* Enhanced cell viability and proliferation, ameliorated mitochondrial oxidative stress and ER stress levels *In vivo:* Promoted regeneration of rat CECs	*IFNGR2* ^a^ *, IRF2* ^a^ *, IRF6* ^a^ & *IRF8* ^a^	Park et al.^81^
	miR-302d-3p	Transient transfection	RPE cell line	Inhibited EMT	*TGFβR2*	Fuchs et al.^22^
	miR-302d—3p	Transient transfection	RPE cells	Inhibited proliferation, migration & cell cycle progression	*CDKN1A*	Jiang et al.^29^
Aging-associated fibrosis	miR-302b-3p	Adeno-associated viral transduction	*In vitro:* HK-2 cells, treated by TGF-*β* *In vivo:* induced fibrosis by UUO	Attenuated renal fibrosis	*TGFβR2*	Sun et al.^28^
	miR-302a-3p	Transient transfection	HK-2, exposed to high glucose	Ameliorated renal fibrosis	*ZEB1* ^a^	Tang et al.^82^
	miR-302/367 cluster	Systemic injection using neutral lipid emulsion	MI mouse model generated by LAD ligation	Promoted fibrotic repair	*Mst1, Lats2 & Mob1b*	Tian et al.^83^
	miR-302b-, c-3p	local injection using hyaluronic acid hydrogel	MI mouse model generated by LAD ligation	Induced cardiac regeneration after MI	ND	Wang et al.^84^
	miR‐302c-3p	Lentiviral transduction	*In vitro:* HMRSV5, treated by TGF‐β1 *In vivo:* High glucose peritoneal dialysate in mice	Alleviated fibrosis	*CTGF* ^a^	Li et al.^85^
	miR-302c-3p	Transient transfection	LX-2 cells treated by TGF-*β*; HSCs from the livers obtained from CCL4-injected mice	Attenuated hepatic fibrogenesis	*E6AP*	Kim et al.^86^
	miR-302a-, b-, d-3p	hiPSC-derived exosomes	*In vitro:* HSCs, treated by TGF-β *In vivo:* Induced liver fibrosis by CCl4 and bile duct ligation in mice	Mitigated fibrosis	ND	Povero et al.^87^
	miR-302a-3p	hiPSC-derived exosomes	Induced pulmonary fibrosis by bleomycin in mice	Alleviated fibrosis	*TET1*	Zhou et al.^88^
Aging-linked to cellular senescence	miR-302b-3p	hESC-derived exosomes	*In vitro:* LO2 cells, induced senescence by doxorubicin *In vivo:* Naturally aged mice injected with Exos-302b	*In vitro:* Reversed proliferative arrest in senescent cells *In vivo:* Extended lifespan & improved physical ability and cognition in aging mice	*Cdkn1a* & *Ccng2*	Bi et al.^27^
	miR-302b-3p	MSC-derived exosomes	MSC cultured under low oxygen conditions	Delayed senescence & increased stemness	ND	Mas-Bargues et al.^89^
	miR-302d-3p	Transient transfection	hASDC, induced oxidative stress by ROS inducers (CoCl2 & SIN-1)	Inhibited oxidant-induced ROS generation & controlled proliferation and cell survival	*CDKN1A* & *CCL5*	Kim et al.^5^
	miR-302a-, b-, c-, d-3p	Transient transfection	HMEC, induced by retroviral transduction of RasG12V	Rescued cell senescence	*p21*	Borgdorff et al.^21^
Senescence Induction	miR‐302b‐3p	Transient transfection	Induced aging by D‐galactose injection in mice	Augmented skin fibroblast senescence	*JNK2*	Tan et al.^90^
	miR-302a-3p	Transient transfection	H9c2 cells, induced senescence by oxygen and glucose deprivation in medium	Enhanced cardiomyocyte apoptosis	*Mcl-1*	Fang and Yeh^91^

Abbreviations: Aβ: Amyloid-β; ND: non-determined; MPTP: Methyl-4-phenyl-1,2,3,6-tetrahydropyridine; hCEC: Human corneal endothelial cell; RPE: Retinal pigment epithelium; EMT: epithelial-to-mesenchymal transition; UUO: Unilateral ureteral obstruction; MI: Myocardial infarction; LAD ligation: Left anterior descending artery ligation; HMRSV5: Human peritoneal mesothelial; TGFβR2: TGF-β receptor2; HSC: Hepatic stellate cell; CCl4: carbon tetrachloride; Exos-302b: miR-302b-containing exosomes; hiPSC: Human induced pluripotent stem cell; hESC: Human embryonic stem cell; MSC: Mesenchymal stem cell; hASDC: Human adipose tissue-derived mesenchymal stem cell; CoCl2: Cobalt chloride; SIN-1: 3-morpholinosydnonimine hydrochloride; HMEC: Human mammary epithelial cell.

a miRNA target predicted or partially validated.

The therapeutic potential of the miR-302/367 cluster extends prominently to age-related ocular disorders, where declining visual acuity often results from cellular senescence impairing the regenerative capacity of human corneal endothelial cells. In this regard, miR-302a has been shown to support corneal endothelial cell regeneration through multiple mechanisms, including the reduction of interferon-induced autophagy, attenuation of senescence markers, decreased oxidative stress, improved mitochondrial function, and enhanced wound healing capacity.^81^ Moreover, miR-302d-3p represses vascular endothelial growth factor (VEGF), thereby effectively inhibiting TGFβ-induced EMT and VEGFA secretion while promoting the restoration of epithelial phenotypes in “mesenchymalized” retinal pigment epithelial (RPE) cells.^22^ These properties confirm the cluster’s potential for treating age-related visual disorders such as age-related macular degeneration (AMD). AMD pathogenesis involves RPE cell dedifferentiation, and miR-302d-3p has been identified as a key regulator of RPE cell behaviors, including migration, invasion, and differentiation, through its targeting of the *P21* gene.^29^ These findings suggest that therapeutic strategies utilizing miR-302/367 cluster modulation may offer great promise for slowing or reversing AMD progression.

### Anti-aging effects of miR-302/367 cluster in tissue fibrosis

The miR-302/367 cluster has been frequently reported to exert anti-senescence effects in various fibrosis models. In diabetic kidney disease (DKD) models, miR-302a-3p overexpression effectively suppresses EMT, a key mechanism driving renal fibrosis. Mechanistically, miR-302a-3p downregulates the transcription factor ZEB1 and modulates E-cadherin and vimentin expression.^82^ Moreover, ectopic expression of miR-302b-3p improves renal fibrosis by directly targeting TGFβR2, consequently inhibiting downstream TGF-β/Smad signaling.^28^ In hepatic models, miR-302c-3p exerts anti-fibrotic activity by targeting the E6AP protein, thereby inhibiting TGFβ-mediated fibrosis in both LX-2 and primary hepatic stellate cells.^86^ For instance, in bleomycin-induced pulmonary fibrosis models, iPSC-derived EVs enriched with miR-302a-3p significantly attenuated fibrosis by reducing collagen deposition. Mechanistically, miR-302a-3p directly targets *TET1*, resulting in the suppression of M2-type macrophages, a critical pathway in fibrotic progression.^88^

Using in vitro and in vivo models of peritoneal fibrosis, it has been shown that lentiviral delivery of miR-302c-3p reverses the fibrosis-associated increase in the levels of E-cadherin and CTGF, leading to the upregulation of the mesenchymal markers α-SMA and collagen I.^85^ The observation that a miR-302 family member promotes the mesenchymal phenotype to ameliorate a diseased state—contrary to its typical role in promoting the epithelial phenotype during pluripotency—is intriguing. This indicates that the miRNA cluster, with its broad spectrum of gene targets, differentially regulates specific subsets of these targets depending on the cellular context. The net effect of this context-dependent regulation is that miR-302 miRNAs can induce different, and sometimes opposing, phenotypes across various biological situations to enable efficient fate decision, reprogramming, and/or aging reversal.

The regenerative potential of the miR-302/367 cluster has also been demonstrated in cardiac tissue. Overexpression of this cluster in vivo, achieved through a conditional transgenic mouse model, promoted fibrotic scar repair by enhancing cardiomyocyte proliferation via suppression of the Hippo signaling pathway.^83^ However, this expression approach requires further optimization, as long-term overexpression of the miRNA cluster resulted in a dedifferentiated cardiomyocyte phenotype and impaired cardiac function. Notably, the use of miRNA mimics, instead of viral or other vector-based expression systems, may provide a better strategy for miR-302-based cardiac regeneration, at least in some contexts. In support of this, local and sustained delivery of miR-302b- and -302c-3p mimics using a hyaluronic acid hydrogel promoted regeneration of damaged cardiac tissue following ischemic injury.^84^ Collectively, these findings indicate that the miR-302/367 cluster plays an important role in alleviating fibrosis during aging processes by suppressing EMT and reducing profibrogenic markers, thereby enhancing tissue regeneration across multiple tissues.

### The miR-302-mediated restoring of functionality in senescent cells

The rejuvenating functions of the miR-302/367 cluster is particularly evident in its ability to restore functionality in senescent cells. In human mammary epithelial cell senescence models, miR-302a-3p overexpression was found to effectively reverse RasG12V-induced senescence by downregulating *P21*.^21^ Similarly, in human adipose tissue–derived mesenchymal stem cells (MSCs), the overexpression of miR-302a, -302 b, -302c, and -302d markedly promoted MSC survival and decreased oxidative stress damage through the silencing of *CDKN1A* and *CCL5*,^5^ highlighting its potential in maintaining cellular function under stress conditions. Supporting these observations, miR-302b-3p has been identified in hESC-derived exosomes to restore proliferative capacity in senescent cells and reverse senescence phenotypes in both in vitro and in vivo models by targeting the cell cycle inhibitors *CDKN1A* and *CCNG2*.^27^ These results indicate that major cell cycle–related gene transcripts are key targets frequently regulated by miR-302 family members to promote both pluripotency in stem cells and functionality in senescent cells.

Administration of miR-302b-3p in aging mice resulted in the mitigation of progressive weight loss and a 15.4% increase in median lifespan, with both male and female mice exhibiting statistically significant improvements in longevity parameters and reduced mortality risk. Notably, no adverse safety outcomes were reported during the 24-month observation period.^27^ Furthermore, extracellular vesicles (EVs) derived from non-senescent MSCs demonstrated substantial potential to counteract cellular senescence, an effect primarily attributed to their delivery of miR-302b-3p, upregulation of HIF-1α, and reduction in mitochondrial oxidative respiration, collectively enhancing cellular functionality and ameliorating senescence-associated phenotypes.^89^ Therefore, the miR-302/367 cluster appears to regulate several aging-related processes through similar mechanisms, particularly by targeting genes involved in fibrosis, oxidative stress, cell cycle regulation, cellular proliferation, and senescence (Figure 2). Collectively, these studies indicate the remarkable potential of the miR-302/367 cluster to reverse cellular senescence and restore tissue function across diverse biological systems, highlighting its therapeutic potential for age-related conditions. It should be noted that a minority of studies report a context-dependent role for miR-302, linking its elevated expression to senescence in specific models such as d-galactose-induced skin aging and hypoxia-induced cardiomyocyte apoptosis.^90^^,^^91^ The reasons for these divergent findings are not yet clear but may relate to differences in senescence induction methods, expression levels, or cell-type-specific effects. Resolving these discrepancies will require further investigation to define the therapeutic window of miR-302 expression that maximizes rejuvenative benefits while minimizing potential adverse effects.

## Overcoming senescence: miR-302/367 cluster as a dual-function regulator of reprogramming and rejuvenation

Cellular senescence represents a significant barrier to somatic cell reprogramming, greatly suppressing iPSC generation.^20^ In this regard, the miRNAs miR-302a, -302 b, -302c, and -302d have emerged as potent suppressors of senescence pathways, particularly through inhibiting key mediators like *p16INK4a*, *p21*, and *p53*. During reprogramming-induced senescence, the miR-302 family members effectively prevent the upregulation of *p21* and *p130*, thereby overcoming this critical hurdle to cellular rejuvenation.^20^ As reviewed throughout this article, the miR-302/367 cluster counteracts many hallmarks of aging. Its dual function in promoting pluripotency and inhibiting senescence positions the miR-302/367 cluster as a safe and powerful tool for regenerative medicine applications. Its properties make it particularly suitable for partial reprogramming-based rejuvenation, as discussed in the following.

The discovery of partial reprogramming—using transient expression of Yamanaka factors (OSKM)—has opened new avenues for rejuvenating senescent cells without fully erasing cellular identity. This approach repairs age-related damage and resets epigenetic clocks while avoiding tumorigenic risks associated with the complete induction of pluripotency. In this regard, reprogramming factors have been reported to mitigate mesenchymal drift observed in human biopsies from various age-related diseases, ameliorating age-associated changes by restoring epigenetic and transcriptomic signatures to a more youthful state, without inducing tumor formation.^93–95^ Several studies have also demonstrated that partially reprogrammed cells acquire progenitor-like states, with downregulated differentiation markers and restored regenerative potential.^96–99^ However, since viral/vector approaches raise risks of genomic integration and other concerns, safer nonintegrative strategies based on miRNAs, in particular the miR-302/367 cluster, combined with small-molecule compounds may offer a more viable, tunable alternative for inducing and sustaining rejuvenation. Notably, small-molecule chemicals that substitute for Yamanaka factors have shown remarkable potential in the reversal of aging-related features and the promotion of tissue regeneration both in vitro and in vivo. In this regard, chemical reprogramming has achieved transcriptomic age reversal in human somatic cells in just a week while preserving original cell identity and reducing senescence markers.^100^ Schoenfeldt et al. reported that a short-term exposure to a two-chemical (RepSox and Tranylcypromine) treatment can reverse certain aging hallmarks in fibroblasts and keratinocytes. Importantly, this small-molecule cocktail ameliorated senescence signatures and enhanced both lifespan and reproductive longevity in *Caenorhabditis elegans* as an in vivo model.^101^ On the other hand, given its well-established role in iPSC generation,^17–19^ the miR-302/367 cluster represents a promising alternative approach to partial reprogramming. Since the miR-302/367 cluster (i) is a potent reprogramming agent (reviewed in a previous study^14^) and (ii) promotes adult cardiomyocyte proliferation and heart regeneration when overexpressed following experimental myocardial infarction,^83^ it may serve as a safer alternative to OSKM-based partial reprogramming, which has also been shown to alleviate myocardial damage.^96^ In contrast to OSKM-mediated heart regeneration, miR-302-mediated cardiac regeneration is less likely to give rise to heart tumor formation, as potent overexpression of the cluster has been reported to induce cell death in abnormally reprogrammed cells.^18^ However, this advantage may have a dark side and be detrimental to tissue regeneration itself when the miR-302/367 cluster is persistently expressed. This concern may be addressed by tissue-targeted delivery of the cluster or the short-term systemic administration of individual miRNA mimics derived from the miR-302/367 cluster.

The miR-302/367 cluster-induced partial reprogramming offers several advantages. First, it can be delivered in an integration-free manner as a precursor miRNA transcript harboring all the family members. Second, it can be efficiently delivered using non-viral vectors due to its small molecular size. Third, it mitigates the risk of tumorigenicity by substituting for the oncogenic factor c-Myc and suppressing oncogenic signaling pathways involving AKT1 and BMI-1.^16^^,^^47^ Fourth, the application of a single member from this cluster may serve as a nearly complete representative of the entire cluster, exerting clinically sufficient regenerative and anti-aging effects.

Besides its safety profile, miR-302 miRNAs promote genomic stability in cells by reducing DNA damage,^21^^,^^22^^,^^83^ a common occurrence in stressed or senescent cells.^20^^,^^21^^,^^51^ On the other hand, small molecules upregulating the miR-302/367 cluster (eg, sodium butyrate) or targeting epigenetic regulators (eg, HDAC2 inhibitors like VPA) synergize with the miR-302/367 cluster^19^^,^^59^^,^^102–104^ by providing a permissive chromatin state for partial reprogramming.

Cellular processes driving tissue repair and rejuvenation can also lead to tumorigenesis if not precisely regulated.^105^ The miR-302/367 cluster has been reported to have a dual role in cancer, functioning both as a tumor suppressor (more frequent) and as an oncogene (less frequent) depending on tumor type and cellular context.^106–109^ This duality may be influenced by expression levels, where the cluster promotes a stem-like state at lower levels but can trigger tumor-suppressive cell cycle arrest when highly expressed. In glioblastoma, EVs secreted from miR-302/367-expressing cells efficiently delivered the miRNA cluster to the glioblastoma cancer stem–like cells, inhibiting their proliferation and in vivo tumorigenesis.^106^ Similarly, in cervical carcinoma, the ectopic expression of the miR-302/367 cluster potently suppressed proliferation and tumor formation by downregulating AKT1 and Cyclin D1, thereby inducing a G1/S cell cycle blockade.^110^ This tumor-suppressive function is reinforced by its ability to inhibit the tumorigenicity of human PSCs through the coordinate suppression of the CDK2 and CDK4/6 cell cycle pathways.^48^ In breast cancer, the miR-302 family acts as a potent tumor suppressor by cooperatively sensitizing cells to chemotherapy via suppression of P-glycoprotein^111^ and by directly targeting ATAD2 to inhibit proliferation, migration, and invasion.^112^ Conversely, in germ cell tumors, the miR-302/367 cluster is reported to be upregulated and serve pro-tumor functions.^107^^,^^108^ In prostate cancer, the cluster forms a regulatory axis with LATS2/YAP signaling pathway to promote cancer cell growth.^109^ This suggests that the oncogenic function of the miR-302/367 cluster is less common and likely tied to very specific cellular contexts.

Notably, this cluster has been shown to promote pluripotency while simultaneously suppressing tumorigenic risk,^47^^,^^48^ highlighting its capacity to balance regenerative benefits with tumor-suppressive control. Therefore, further research is required to elucidate the precise regulatory mechanisms governing its function in regenerative medicine and cancer therapy. This remains a critical area for future investigation, specifically to define the therapeutic window where miR-302/367 expression promotes rejuvenation and stemness without inadvertently facilitating oncogenesis.

## The miR-302/367 cluster: a promising therapeutic tool in regenerative medicine

**Figure 3.**
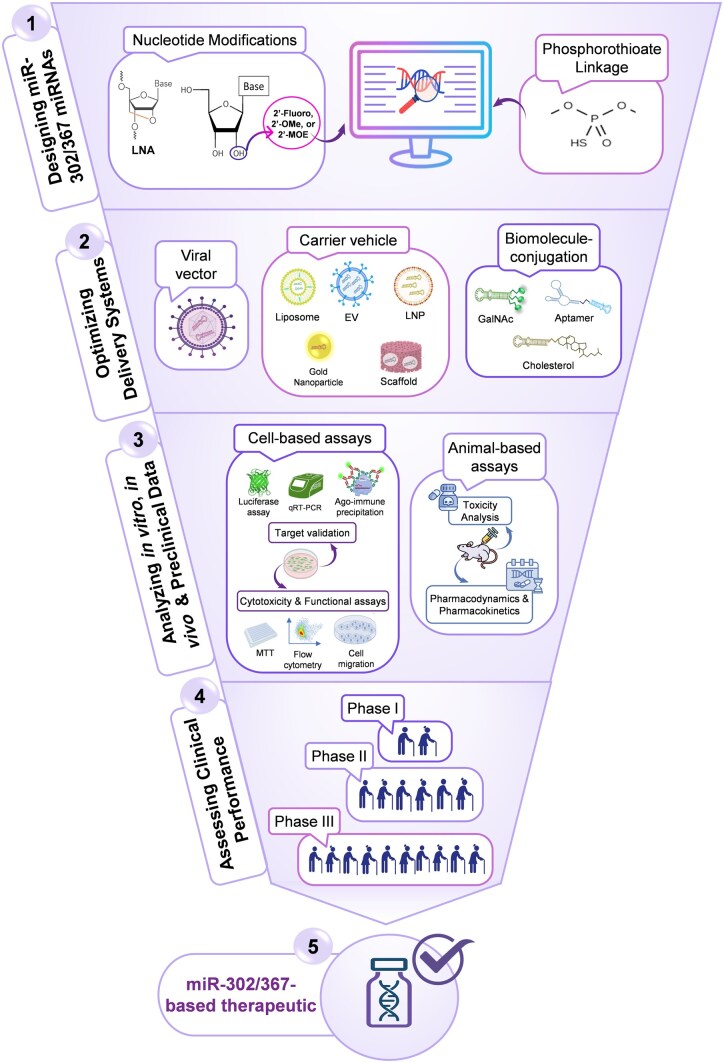
Development process for translating the miR-302/367 cluster into a market-approved therapeutic. The process involves several key stages. First, the therapeutic sequence must be designed, paying attention to key considerations: rendering the passenger strand inactive while maintaining guide strand activity, ensuring the sequence does not function as an anti-miRNA. During design, phosphorothioate linkages and chemical modifications are applied to enhance stability and specificity. Second, the most suitable delivery system must be selected to ensure efficient delivery while minimizing side effects. Third, comprehensive preclinical analysis is conducted, including in vitro and in vivo studies to validate targets, assess toxicity, and evaluate pharmacokinetics and pharmacodynamics in cellular and animal models to establish therapeutic potential. Fourth, phased human trials (Phases I–III) are conducted to confirm safety and efficacy, culminating in regulatory approval for clinical use. Ago: Argonaute.

The miR-302/367 cluster holds promising therapeutic potential to restore cellular function and improve disease outcomes. As with other therapeutic miRNA applications, multiple critical steps (Figure 3) must be effectively completed for miR-302-based therapies to reach the market; however, this process is challenged by three main factors that also apply to other therapeutic miRNAs: efficient delivery, metabolic stability, and the risk of toxicity.^113–115^ We discuss each of these challenges and opportunities.

Regarding stability, chemical nucleotide modifications (eg, locked nucleic acids [LNAs], 2ʹ-O-methylation [2ʹ-OMe], 2-methoxyethyl [2′-MOE], 2ʹ-fluoro), as well as substituting phosphodiester linkages with phosphorothioate bonds can significantly enhance in vivo miRNA stability.^116^^,^^117^ These modifications are widely employed in the design of miRNA-based therapeutic sequences (Table 3). Although LNA nucleotides have been frequently employed in the design of anti-miRNA therapeutics due to their nuclease resistance and high binding affinity,^123^^,^^125^^,^^127–129^^,^^131^ they have only recently begun to be incorporated into miRNA mimic design strategies in research studies.^132–135^ This modification provides enhanced target specificity through improved guide strand selection efficiency, minimizing off-target effects.

**Table 3.** Summary of therapeutic miRNA-based clinical trials.

	miRNA drug name	Modifications	Delivery	Type of disease	Clinical trial Phase	Administration	Reference
miRNA mimics	Remlarsen (MRG-201) (miR-29b-3p)	2′‐OMe	Cholesterol conjugation	Fibrotic scar	Phase II (Discontinued)	Intradermal	Gallant-Behm et al.^118^ NCT03601052
	MesomiR (miR-16-5p)	2′‐OMe	Nonliving bacterial minicells conjugated by EGFR-specific antibody	Recurrent malignant pleural mesothelioma (MPM) & non-small cell lung cancer (NSCLC)	Phase I (Completed)	Intravenous	Reid et al.^119^ NCT02369198
	MRX34 (miR-34a-5p)	Not mentioned	Liposome nanoparticle	Primary liver cancer, lymphoma, melanoma, renal cell carcinoma, NSCLC, SCLC	Phase I (Terminated)	Intravenous	Hong et al.^120^ NCT01829971
	TenoMiR (miR-29a-3p)	2′-Fluoro and 2′‐OMe	Cholesterol conjugation	Lateral epicondylitis	Phase II (Completed)	Intratendinous	Millar et al.^121^ NCT06192927
	INT-1B3 (miR-193a-3p)	Not mentioned	LNP	Advanced solid tumors	Phase I (Terminated, insufficient funding)	Intravenous	Kotecki et al.^122^ NCT04675996
Anti-miRNAs	CDR132L (miR-132-3p)	LNA, PS	Not employed	Reduced left ventricular ejection fraction after myocardial infarction (HF-REVERT)	Phase II (Completed)	Intravenous	Bauersachs et al.^123^ NCT05350969
	Lademirsen (miR-21-5p)	2′-MOE, PS	Not employed	Alport syndrome	Phase II (Terminated)	Subcutaneous	Gale et al.^124^ NCT02855268
	MRG-110 (miR-92a-3p)	LNA	Not employed	Cardiovascular disease & wound healing	Phase I (Completed)	Intravenous	Abplanalp et al.^125^ NCT03603431
	RG-125/AZD4076 (miR-103-3p/107-3p)	Not mentioned	GalNAc conjugation	Non-alcoholic steatohepatitis (NASH) in patients with type 2 diabetes/prediabetes	Phase I (Completed)	Subcutaneous	NCT02826525
	RG-101 (miR-122-5p)	Not mentioned	GalNAc conjugation	Chronic hepatitis C infection	Phase II (Completed)	Subcutaneous	van der Ree et al.^126^ EudraCT: 2013-002978-49
	MRG-106 (Cobomarsen, miR-155-5p)	LNA	Not employed	Cutaneous T-cell lymphoma (CTCL) & Mycosis fungoides	Phase II (Terminated)	Intravenous	James et al.^127^ NCT03837457
	Miravirsen (SPC3649, miR-122-5p)	α-D-oxy-LNA, PS	Not employed	Chronic hepatitis C virus	Phase I (Completed)	Subcutaneous	Ottosen et al.^128^ NCT02452814
	LNA-i-miR-221 (miR-221-3p)	LNA, PS	Not employed	Refractory-MM & advanced solid tumors	Phase I (Completed)	Intravenous	Tassone et al.^129^ NCT04811898
	TTX-MC138 (miR-10b-5p)	Not mentioned	Iron oxide nanocarrier	Advanced solid tumors	Phase I (Completed)	Intravenous	Medarova et al.^130^ NCT05908773
	RGLS4326 (miR-17-5p)	LNA, 2ʹ-fluoro, 2ʹ-OMe, PS	Not employed	Polycystic kidney disease	Phase I (Completed)	Subcutaneous	Lee et al.^131^ NCT04536688

Abbreviations: 2′‐OMe: 2'-O-methylation; LNP: lipid nanoparticle; LNA: locked nucleic acid; PS: phosphorothioate linkage; 2′-MOE: 2-methoxyethyl; NE: not employed.

With respect to in vivo miRNA delivery, the miR-302/367 cluster or its individual miRNAs can be delivered using vectors or synthetic miRNA mimics, respectively. Viral vectors provide high transduction efficiency and sustained expression. To achieve targeted expression of the vectors, tissue-specific promoters, such as the MHCK7 promoter, which was used for the muscle-specific expression of the FDA-approved gene therapy Elevidys,^114^^,^^136^ should be used. Since viral approaches are associated with immunogenicity and other safety concerns, alternative delivery platforms, including lipid nanoparticles (LNPs), polymer-based carriers (eg, polyethylenimines [PEIs], polyethylene glycol [PEG]), inorganic materials (eg, gold nanoparticles), EV-based delivery systems, and molecular conjugation such as targeting mediated by antibodies and triantennary *N*-acetylgalactosamine (GalNAc) have emerged to enable tissue-targeted delivery of small RNAs including miRNAs while protecting therapeutic miRNAs against degradation. LNPs, PEGylation, and GalNAc conjugates have previously been successfully used in multiple FDA-approved oligonucleotide therapeutics and represent promising options as clinically validated miRNA delivery systems. LNPs provide broad applicability through efficient encapsulation, while GalNAc provides precise hepatocyte targeting via the asialoglycoprotein receptor (ASGPR) on hepatocytes. Interestingly, PEGylation of oligonucleotides enhances stability, prolongs circulation half-life, and reduces immunogenicity.^137–140^ As shown in Table 3, within miRNA-based clinical trials, RG-101 and RG-125 employed GalNAc-conjugated delivery strategies for liver-based diseases, whereas INT-1B3 is delivered using LNPs.^122^^,^^126^

EVs serve as an alternative delivery vehicle for the therapeutic miR-302/367 cluster. PSC-derived exosomes containing miR-302b-3p have shown promise for aging interventions, including lifespan extension and alleviation of liver fibrosis.^30^^,^^99^ While LNPs and EVs are not as tissue specific as GalNAc conjugates, other targeted systems for the delivery of miR-302/367 cluster members (eg, using senescent cell-targeting peptides) may provide a more precise therapeutic approach to aging reversal by enhancing rejuvenation effects while protecting healthy tissues. Consistent with this, EVs derived from MSCs conjugated to a cardiomyocyte-targeting peptide and loaded with miR-302d-3p were taken up more effectively by cardiomyocytes, enhanced cardiomyocyte proliferation in vitro, suppressed myocardial apoptosis and inflammatory responses, and promoted cardiac function in vivo.^141^ Another promising approach to achieve local and sustained release of therapeutic miRNAs is the use of emerging biodegradable scaffold techniques. This approach enabled sustained, localized delivery of miR-302b- and -302c-3p, leading to regeneration after myocardial infarction in mice.^84^ Overall, while emerging delivery methods may advance into clinical trials in the near future, we envision that FDA-approved approaches would currently be the most reliable way of delivering the miR-302/367 cluster in clinical applications.

Cellular toxicity of therapeutic miRNAs is another clinical hurdle. When (over)expressed, a single miRNA can target many mRNA transcripts, potentially disrupting unintended cellular pathways. This effect can cause cytotoxicity, making clinical development of miRNAs more challenging compared to other therapeutic oligonucleotides such as siRNAs. To minimize off-target effects of the miR-302/367 cluster, chemical modifications can be appropriately incorporated during sequence design, and in silico target identification using bioinformatic tools can be further optimized.^114^^,^^142^ Moreover, assessing the immunostimulatory potential of designed sequences (eg, by screening for UG-rich motifs)^143^ is recommended prior to experimental validation. Investigational approaches, including luciferase reporter assays and Argonaute immunoprecipitation, remain the gold standard for identifying and confirming miRNA–target interactions (see Figure 3).^144^^,^^145^ Another critical challenge regarding toxicity and side effects is determining the optimal dosage. Since the activity of miRNAs relies on competition for the RISC complex, miRNA mimic overexpression can compete with endogenous miRNA species and interfere with cellular pathways, thereby causing toxicity.^146^ Therefore, it is critically important to find a dose that is both nontoxic and efficacious for therapeutic administration. In the context of cell fate reprogramming, the dose-dependent effects of miR-302 have been demonstrated, identifying the threshold concentration required for effective reprogramming while avoiding adverse effects,^18^ suggesting that a safe, efficacious dose can be determined for other miR-302-based applications. Therefore, carefully designed preclinical studies conducted after successful in vitro and in vivo assays are crucial for optimizing dosing and evaluating toxicity to ensure safety prior to clinical trials. Additionally, inhibitory or undesired endogenous miRNAs can be partially sequestered using miRNA sponges,^147^ thereby further supporting the intended therapeutic effects of the miR-302/367 cluster by allowing it to sufficiently incorporate into the RISC.

Although successful clinical translation of miR-302 miRNAs requires precise design, careful validation, optimized delivery, and thorough preclinical dose–toxicity examination, confirmation of their safety and efficacy in clinical trials (see Figure 3) is required for their successful clinical translation. The successful completion of these essential steps would enable a revolution in regenerative medicine for age-related disorders with miR-302-based therapies.

## Conclusion

The miR-302/367 cluster connects pluripotency induction with cellular rejuvenation. By concurrently resetting epigenetic clocks, suppressing senescence pathways, and fine-tuning critical signaling networks such as TGF-β and BMP pathways, this miRNA cluster offers an effective therapeutic strategy against age-related decline. The miR-302/367 cluster enhances reprogramming efficiency while reducing risks inherent to conventional approaches, notably through replacing oncogenic factors such as c-Myc and its nonintegrative nature, which establishes it as a safer, more effective option for producing clinically relevant cells.

Integrating miR-302/367 biology with partial reprogramming approaches may provide targeting the aging process. The cluster’s ability to rejuvenate cellular age while maintaining somatic identity could have broad applications from regenerative therapy to systemic rejuvenation. Importantly, individual members (eg, miR-302a or miR-302d) possess similar activity to the entire cluster due to a shared conserved seed sequence and overlapping targets. Delivery of a single miRNA, which is easier, cost-effective, and more pharmacokinetically defined, could facilitate therapeutic development without compromising efficacy. Development of chemically enhanced stable single-miRNA therapeutics may facilitate regulatory approval, without a loss of rejuvenation and regenerative potential of the cluster. Current developments in delivery platforms, particularly EV-based delivery systems, are promising; however, their reliable clinical translation faces two major obstacles: heterogeneity and large-scale production, which necessitates the establishment of standardized manufacturing and quality control protocols. Moreover, major challenges still exist with respect to optimal dosing, tissue-specific targeting efficiency, and the long-term impact of epigenetic reprogramming in aged tissues.

Several key questions must be addressed to advance the therapeutic utility of the miR-302/367 cluster: What are the precise molecular mechanisms underlying its anti-aging activity? How can long-term safety be ensured, particularly regarding the potential tumorigenic risks of partial reprogramming? How can sequence design, chemical modifications, and delivery scaffolds for individual miR-302/367 members be optimized to maximize efficacy and specificity? Furthermore, what are the optimal delivery systems for achieving safe and effective tissue targeting, and to what extent should chemical modifications be applied to individual nucleotides to enhance stability and precision without compromising activity? Thus, the miR-302/367 cluster stands not merely as a subject of scientific inquiry but as a foundational tool with the potential to bridge the long-sought gap between regenerative capacity and the extension of human healthspan.

## Funding 

Research in the H.B. and S.M. labs is supported by funds from the Royan Institute, Tehran, Iran.

## Conflicts of interest 

None declared.
